# Practical and Analytical Chemistry

**Published:** 1892-12

**Authors:** 


PRACTICAL AND ANALYTICAL CHEMISTRY. A Complete Course in Chemical Analysis. By HENRY TRIMBLE, PH. M., Professor of Analytical Chemistry in Philadelphia College of Pharmacy. Fourth edition, with illustrations; pp. 191. Philadelphia: P. Blakiston, Son & Co. 1892.

The publication of four editions of a work on qualitative analysis within seven years is not of common occurence, and speaks well the value of such a book. The author has given us an excellent little work upon practical and analytical chemistry, and we have only words of praisefor the painstaking care exhibited throughout the volume by both the author and the publishers. J. A. M.

